# Molecular detection of *Rickettsia aeschlimannii*, *Borrelia theileri*, and *Francisella-*like endosymbionts in *Camelus dromedarius* and dogs in Luxor, Egypt

**DOI:** 10.1038/s41598-025-91530-x

**Published:** 2025-04-15

**Authors:** Hassan Y. A. H. Mahmoud, Ahmed M. Soliman, Moshera S. Shahat, Ali A. Hroobi, Ali H. Alghamdi, Abdullah M. Almotayri, Tetsuya Tanaka, Walaa F. A. Emeish

**Affiliations:** 1 Division of Infectious Diseases, Animal Medicine Department, Faculty of Veterinary Medicine, South Valley University, Qena, 83523 Egypt; 2 Biotechnology Department, Animal Health Research Institute, Agricultural Research Center, Dokki Giza, 12618 Egypt; 3 Laboratory of Infectious Diseases, Joint Faculty of Veterinary Medicine, Kagoshima University, Kagoshima, 890-0065 Japan; 4 Division of Internal Medicine, Animal Medicine Department, Faculty of Veterinary Medicine, South Valley University, Qena, 83523 Egypt; 5 Department of Biology, Faculty of Science, Al-Baha University, Al-Baha, Saudi Arabia; 6 Laboratory of Animal Microbiology, Graduate School of Agricultural Science, Tohoku University, Sendai, 980-8572 Japan; 7 Department of Fish Diseases, Faculty of Veterinary Medicine, South Valley University, Qena, 83523 Egypt

**Keywords:** Bartonella, Borrelia, Coxiella, Camels, Dogs, Francisella, Rickettsia, PCR, Infectious-disease diagnostics, Parasitology

## Abstract

Vector-borne bacterial pathogens can cause disease in a range of animals, including dromedary camels and dogs, but epidemiological and molecular studies on these pathogens are scarce in southern Egypt. In this study, we screened camels and dogs in southern Egypt (Luxor) for vector-borne bacterial pathogens, with molecular analysis of 200 blood samples collected from camels and dogs in the region. The *Rickettsia aeschlimannii gltA* gene was detected in 5% (5/100) of camel blood samples and 1% (1/100) of dog blood samples. This study is the first report *of Rickettsia aeschlimannii* in camel blood in southern Egypt. Additionally, the *16S rRNA* gene of a *Francisella*-like endosymbiont was detected in both camel and dog blood for the first time, with infection rates of 2% (2/100) in camels and 2% (2/100) in dogs. In dog blood, the *Borrelia theileri flaB* gene was detected for the first time in southern Egypt at a positivity rate of 5% (5/100). Neither Coxiella nor Bartonella species were detected in this study. In southern Egypt, *Rickettsia aeschlimannii*, *Borrelia theileri*, and *Francisella-*like endosymbionts were detected in camels and dogs, providing valuable information about their infection rate and these findings contribute to a better understanding of their transmission dynamics.

## Introduction

There are many common vectors-borne diseases in Egypt^1^. The diseases they transmit include rickettsioses, emerging vector-borne diseases caused by *Rickettsia* species (Gram-negative obligatory intracellular prokaryotic bacteria) that can infect humans and animals^2^. Several *Rickettsia* species are categorized into spotted fever or typhus groups, including *Rickettsia belli groups*, and *Rickettsia candensis* groups^3^. *Rickettsia africae* is Africa’s most common rickettsial species and causes African tick-borne fever in humans^4^. Other rickettsiae such as *Rickettsia. aeschlimannii*, *Rickettsia conorii*, *Rickettsia sibirica mongolitimonae, and Rickettsia massiliae* have been detected in ticks and animals in Africa^5–8^.

*Francisella* species are ubiquitous in many ecosystems, and can be found in hosts, ranging from lagomorphs, rodents, insectivores, carnivores, ungulates, marsupials, birds, and amphibians, to invertebrates^9^. The most widely reported *Francisella* species is *Francisella tularensis*, the highly infectious and zoonotic pathogen that causes tularemia, a potentially fatal disease if untreated*. Francisella tularensis*, its subspecies, and *Francisella-*like endosymbionts share a high nucleotide identity (exceeding 98% similarity on average)^10^. Ticks act as reservoirs for many species of *Francisella*, which often colonize reproductive tissues to facilitate transovarial transmission^11,12^, or the salivary glands, to facilitate transmission through tick bites^13,14^. Furthermore, trans-stadial transmission has also been confirmed^15^.

*Borrelia* is a diverse genus of Gram-negative bacteria that act as obligatory parasites^16–18^*.* A Borrelia species can be transmitted to vertebrates by ticks and other arthropod vectors^16–18^. Some *Borrelia* species are the pathogens behind emerging and reemerging infectious diseases that pose a significant global health concern to both animals and humans^19,20^. *Borrelia theileri* is the causative agent of borreliosis in livestock, and can affect cows, goats, and sheep^21–24^. In Egypt, the data on borreliosis in animal hosts are sparse; only a few studies have reported detection of *Borrelia burgdorferi* and *Borrelia theileri* in hard ticks and *Rhipicephalus annulatus*^25^. In recent years, *Borrelia theileri* has reportedly been detected in tick blood collected from cattle in Argentina and Cameroons, and in sheep blood collected in Algeria^22,26,27^.

Bacteria in the genus Coxiella exhibits the characteristics of a rickettsial pathogen, and was accordingly initially classified as rickettsiae. Like rickettsiae, they are spread by arthropods, replicate intracellularly in eukaryotic cells, and stain weakly with Gram staining^28^. However, Coxiella were found to differ from rickettsiae in that they can also spread by ingestion or inhalation as well as arthropods. The genus Coxiella, along with other genera like Legionella and Francisella, is currently classified in the category Protobacteria, separately to rickettsiae^28^.

There is just one species in the genus Coxiella, *Coxiella burnettii*, a small, rod-shaped, Gram-negative bacteriun that causes coxiellosis in both domestic and wild animals and Q-fever in humans^29^. It is still unclear how ticks contribute to the spread of this disease in animals^30^; however, a number of studies indicate that ticks serve as a reservoir and important vectors for spreading Coxiella in wild mammals^31^.

Bartonella is a Gram-negative, aerobic, short bacillus which shows facultative intracellular infection of mammalian endothelium cells and erythrocytes^32^. Bartonella species can be found in a variety of mammalian hosts as well as the arthropods that naturally circulate between them^33^. Bartonellosis infection rates in wild mammals are extremely high^34^. Blood-sucking arthropods, including fleas, lice, ticks, and mosquitoes, can spread this bacterium to humans and other mammals, and it is typically transmitted through flea and lice feces, mosquito bites, and probably tick bites^35^.

Bartonella species and *Coxiella burnetii* are reportedly present in Egypt in both healthy people and those suffering from pyrexia or culture-negative endocarditis^36^. Several areas in the Middle East including Algeria, Tunisia, and Saudi Arabia, have reported vector-borne diseases in domestic carnivores^37–39^. In Egypt, reported investigations on Bartonella species and *Coxiella burnetii* in dogs and camels are limited^40^.

Our goal in this study to survey these two divergent domestic animal populations for vector-borne pathogens, including Rickettsia, Borrelia, Francisella-like endosymbionts, Bartonella, and *Coxiella burnetii* using molecular techniques.

## Methods

### Study design, research area, and collection of samples

**Fig. 1:**
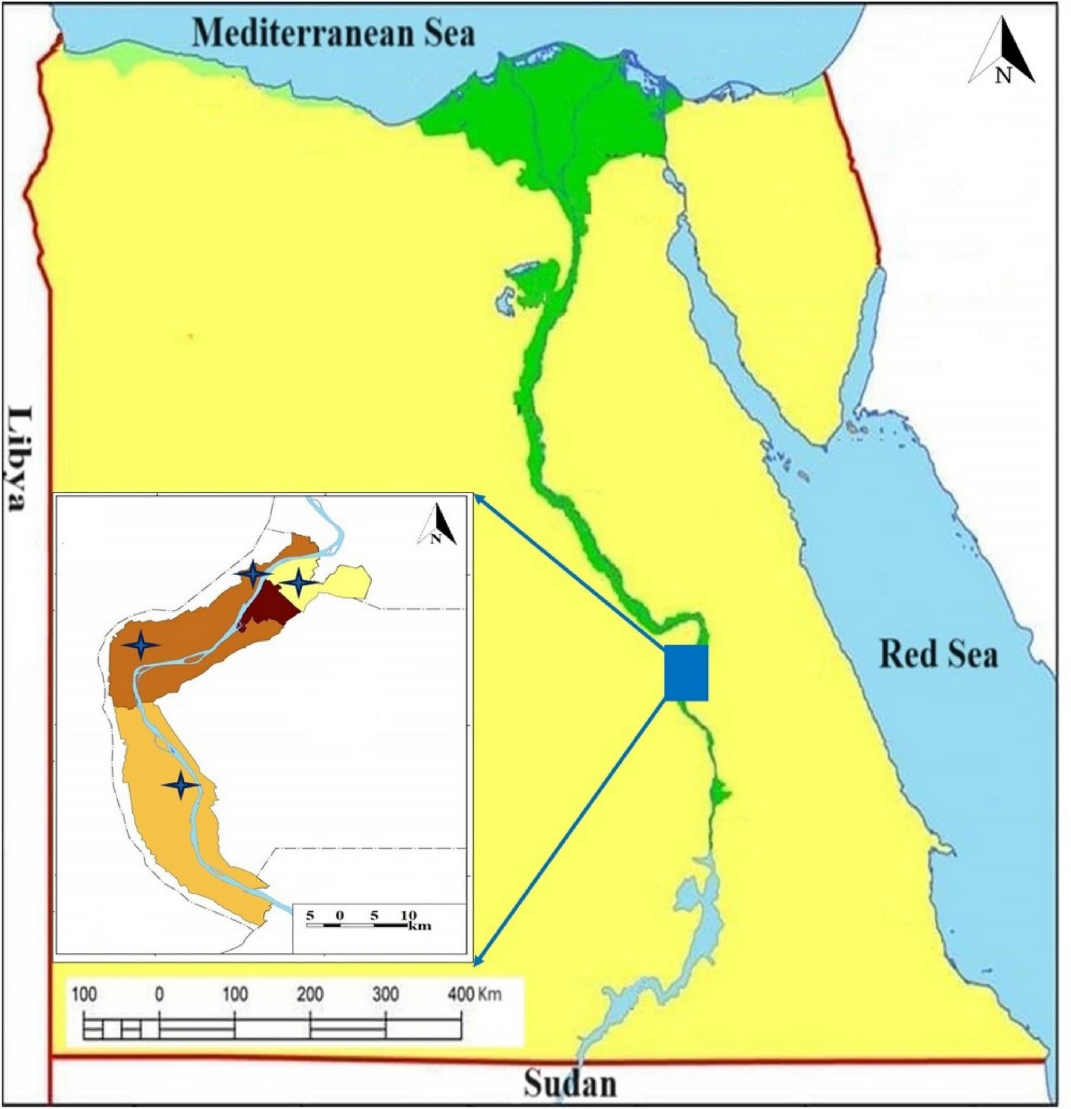
Map of Egypt indicating the study areas where samples were collected from four different regions in Luxor. The blue star represents the collection of samples sites in the present study.

Procedures were performed in accordance with relevant guidelines and regulations, and experimental protocols were approved by the Ethical Research Committee of South Valley University, Faculty of Veterinary Medicine (Approval No. 19/11.08.2021). This study followed relevant guidelines on animal research (Reporting of In Vivo Experiments [ARRIVE]). Camel and dog interactions are frequent in the study area, especially when animals are transported and feed outside. A study was conducted in Luxor (25° 41′ 14.0748’' N 32° 38′ 22.6896’' E) for the detection of vector-borne bacterial pathogen infections in local camel and dog breeds. The study was performed between January 2022 and January 2023 (Fig. 1). There were 100 camels (60 males and 40 females) and 100 dogs (55 males and 45 females) ranging in age from one to four years, and the samples taken from the animals in this study were from small-scale owners, with one or two animals per owner. Most animals were infested with ticks. Whole blood samples for PCR amplification were collected from the jugular or cephalic vein of each animal using sterile, clean vacutainer tubes containing EDTA, and the samples were then stored at − 20 °C until use. Based on the cooperation of animal owners and the accessibility of sampling locations, we selected the number of samples and specific populations and groupings.

### Clinical examination of animals

Before collecting samples, all animals were clinically evaluated for age, mucous membranes, respiratory rate, pulse rate, and body temperature. No obvious clinical abnormalities were detected. Most camels were infested with ticks, ranging from one to ten ticks per animal. On the other hand, only a few dogs were infested with ticks, with just one or two ticks per animal.

### Pathogen detection by PCR

As a first step, we extracted DNA from 200 blood samples using a DNA extraction kit (Wizard® Genomic DNA Purification Kit, USA). The extracted DNA was then subjected to concentration measurement using a Nanodrop 2000 spectrophotometer (Thermo Scientific, Waltham, Massachusetts, USA). PCR was then performed using forward and reverse primers specific for the *citrate synthase* (*gltA*) gene of Rickettsia species^41^, the *16S rRNA* gene of Francisella species^42^, the *Flagellin B* (*flaB*) gene of Borrelia species^43^*, outer membrane protein* (*com1*) gene for Coxiella species^44^, and the *Filamenting temperature-sensitive mutant* Z (*ftsZ*) gene for Bartonella species (Tables 1 and 2)^45^. Tks Gflex DNA Polymerase (TaKaRa, Shiga, Japan) was used in the PCR reaction, which was conducted at a total volume of 10 µL using 5 µL of 2 × Gflex PCR buffer and 0.5 µL of Tks Gflex DNA polymerase (TaKaRa), 0.5 µL of each of the forward and reverse primers with a concentration of 10 µM, 3 µL of nuclease-free water, and 0.5 µL of the template (DNA template with a concentration ranging from 10 to 30 ng/µL). Negative controls containing nuclease-free water were used as negative samples. PCR products were electrophoresed in 1.5% agarose gel in 1 × Tris–acetate-EDTA (TAE) buffer with an electrophoresis device, Mupid (Mupid Co., Ltd., Tokyo, Japan). Gel bands were visualized using a gel documentation system UV device, WUV-M20 (ATTO Co., Ltd., Tokyo, Japan) after staining with 5 g/ml ethidium bromide in 1 × TAE.

**Table 1:** The primers used for the amplification of target fragments of genes in the present study.

Organism	Target gene	Sequence (5′-3′)	Expected size (bp)	References
*Rickettsia* species	gltA*	ATGACCAATGAAAATAATAAT CTTATACTCTCTATGTACA GGGGGCCTGCTCACGGCGG ATTGCAAAAAGTACAGTGAAC	341	^41^
*Francisella* species	*16S rRNA*	GCCCATTTGAGGGGGATACC GGACTAAGAGTACCTTTTTGAGT	1151	^42^
*Borrelia* species	flaB*	GATCA(G/A)GC(T/A)CAA(C/T)ATAACCA(A/T)ATGCA AGATTCAAGTCTGTTTTGGAAAGC GCTGAAGAGCTTGGAATGCAACC TGATCAGTTATCATTCTAATAGCA	344	^43^
*Coxiella* species	com1*	AGTAGAAGCATCCCAAGCATTG TGCCTGCTAGCTGTAACGATTG GAAGCGCAACAAGAAGAACAC TTGGAAGTTATCACGC AGTTG	438	^44^
*Bartonella* species	ftsZ*	GCCTTCAAGGAGTTGATTTTGTTGTTGCCAAT ACGACCCATTTCATGCATAACAGAAC	569	^45^

*gltA: *citrate synthase* gene, *flaB*: *Flagellin B* gene, *com1*: *outer membrane protein* gene*, ftsZ*: *Filamenting temperature-sensitive mutant Z*.

**Table 2:** PCR conditions for the amplification of target fragments of genes.

Organism	Target gene	PCR condition
*Rickettsia* species	gltA*	(95 °C)/(10 min) → [(94 °C)/(30 s)-(52 °C)/(30 s)-(72 °C)/90 s] 30 × → (72 °C)/( 5 min) → 10 °C → ∞ (95 °C)/( 5 min) → [(94 °C)/(30 s)-(52 °C)/(30 s)-(72 °C)/30 s] 30 × → (72 °C)/( 5 min) → 10 °C → ∞
*Francisella* species	16S rRNA*	(95 °C)/( 5 min) → [(94 °C)/(30 s)-(60 °C)/(30 s)-(72 °C)/60 s] 40 × → (72 °C)/(10 min) → 10 °C → ∞
*Borrelia* species	flaB*	(95 °C)/( 5 min) → [(94 °C)/(30 s)-(55 °C)/(30 s)-(72 °C)/30 s] 40 × → (72 °C)/( 5 min) → 10 °C → ∞ (95 °C)/( 5 min) → [(94 °C)/(30 s)-(55 °C)/(30 s)-(72 °C)/30 s] 40 × → (72 °C)/( 5 min) → 10 °C → ∞
*Coxiella* species	Com1*	(95 °C)/( 5 min) → [(94 °C)/(30 s)-(54 °C)/(30 s)-(72 °C)/30 s] 36 × → (72 °C)/( 5 min) → 10 °C → ∞ (95 °C)/( 5 min) → [(94 °C)/(30 s)-(45 °C)/(30 s)-(72 °C)/30 s] 36 × → (72 °C)/( 5 min) → 10 °C → ∞
*Bartonella* species	ftsZ*	(95 °C)/( 5 min) → [(94 °C)/(30 s)-(55 °C)/(40 s)-(72 °C)/60 s] 40 × → (72 °C)/(10 min) → 10 °C → ∞

*gltA: *citrate synthase* gene, *flaB*: *Flagellin B* gene, *com1*: *outer membrane protein* gene, *ftsZ*: *Filamenting temperature-sensitive mutant Z*.

### Sequence and data analysis

Sequence analysis to detect Rickettsia species *gltA, Francisella-*like endosymbiont* 16S rRNA, and Borrelia theileri flaB* were performed using PCR with 50 µL mixtures. PCR amplicons were purified with NucleoSpin Gel and a PCR Clean-Up Kit (Macherey–Nagel, Leicestershire, Germany). After the bi-directional sequences were first examined, the forward and reverse sequences were aligned in MEGA X software to provide sequences for additional examination. The acquired sequences were matched to reference sequences in the GenBank database to verify their identity. Phylogenetic trees were constructed using the maximum Likelihood approach based on the chosen model, and the bootstrap values based on 1000 repeats were used to analyze the phylogenetic trees in order to determine how robust the tree topology^46,47^.

## Results

The overall infection rates for vector-borne bacterial pathogens in this study were 7% (7/100) in camels and 8% (8/100) in dogs (Table 3). In camels, the positivity rate for Rickettsia species was 5% (5/100), with three cases in males and two in females. On the other hand, Francisella species showed a positivity rate of 2% (2/100) and was detected only in female camels. In dogs, the positivity rates of Rickettsia, Francisella, and Borrelia species were 1% (1/100), 2% (2/100), and 5% (5/100), respectively. Rickettsia species was only detected in a male dog, while Francisella species were detected only in two female dogs. Borrelia species were detected in three females and two males (Table 3).

**Table 3:** The infection rates of vector-borne bacterial pathogens in the present study.

Animals	Camels					Dogs		
Organisms	Number of examined animals	Infected number and perecent of infection	Sex		Number of examined animals	Infected number and perecent of infection	Sex	
			Female	Male			Female	Male
*Rickettsia* species	100	5 (5%)	2 (2%)	3 (3%)	100	1 (1%)	0 (0%)	1 (1.0)
*Francisella-like endosymbionts*	100	2 (2%)	2 (2%)	0 (0%)	100	2 (2%)	2 (2%)	0 (0%)
*Borrelia theileri*	100	0 (0%)	0 (0%)	0 (0%)	100	5 (5%)	3 (3%)	2 (2%)
*Coxiella* species	100	0 (0%)	0 (0%)	0 (0%)	100	0 (0%)	0 (0%)	0 (0%)
*Bartonella* species	100	0 (0%)	0 (0%)	0 (0%)	100	0 (0%)	0 (0%)	0 (0%)
Total	100	7 (7%)	4%	3%	100	8 (8%)	5 (5%)	3(3%)

**Fig. 2:**
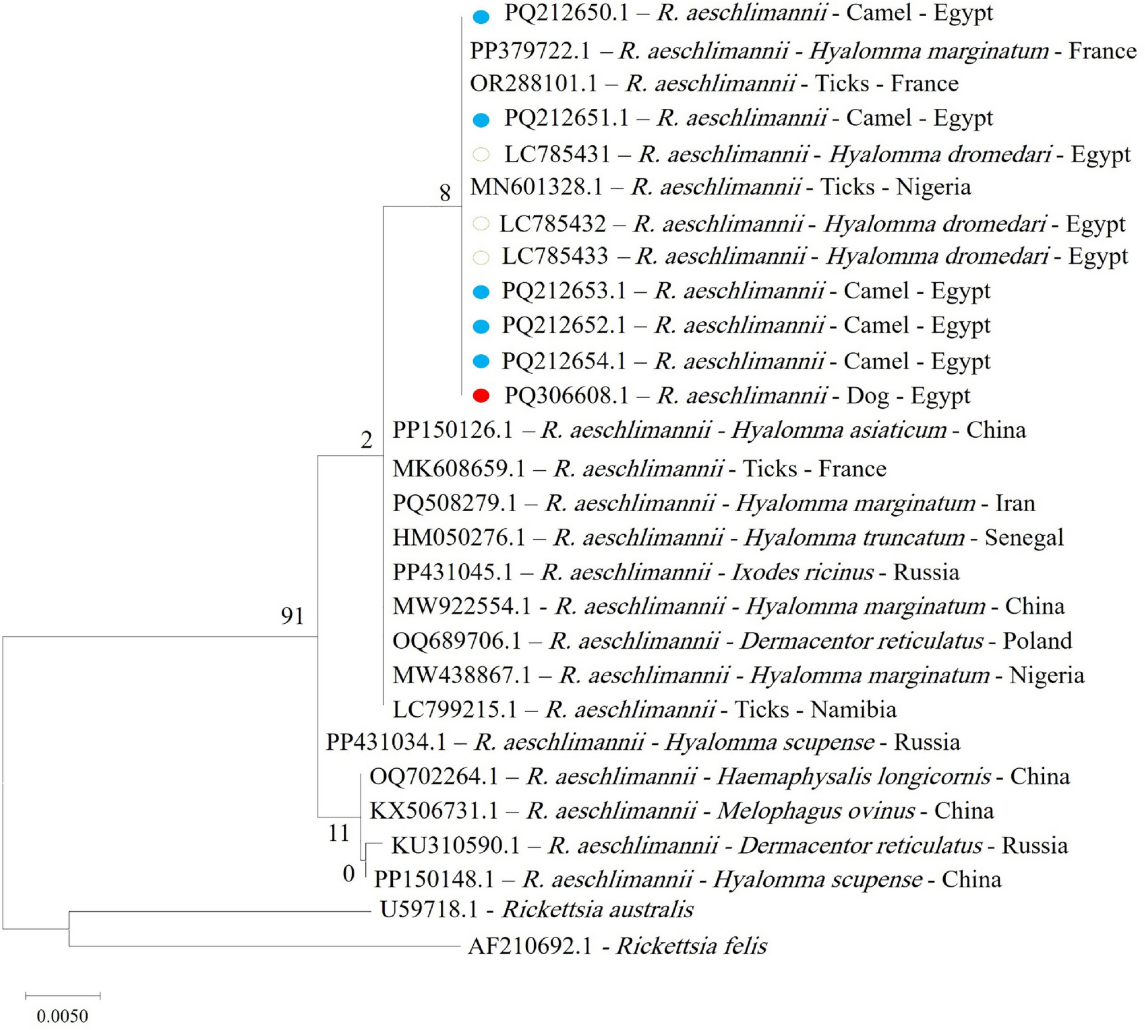
Phylogenetic relations of *Rickettsia aeschlimannii* in camels and dogs, obtained via the maximum-likelihood method and the Kimura three-parameter model based on *citrate synthase* (*gltA*) gene sequences. The percentage of trees on which the related taxa are clustered together is displayed next to the branches. Branch lengths are expressed in terms of the number of substitutions per site, and the tree is drawn to scale. The green unshaded circles related to *Rickettsia aeschlimannii* from *Hyalomma dromedari* in Egypt. The blue shaded circles represent *Rickettsia aeschlimannii* in camels and the red shaded circle represents *Rickettsia aeschlimannii* in dogs in the present study**.**

Eleven PCR products were obtained from 15 positive samples (n = 6 for camels; n5 for dogs) were sent for sequencing. Sequence analysis for *Rickettsia aeschlimannii* was successfully performed on six samples (five from camels and one from a dog). The camel-derived sequences were deposited in the GenBank database with accession numbers from PQ212650.1 to PQ212654.1, sequentially, and the accession number of the dog sample was PQ306608.1. A phylogenetic tree was constructed to elucidate evolutionary relationships among various Rickettsia species (Fig. 2).

**Fig. 3:**
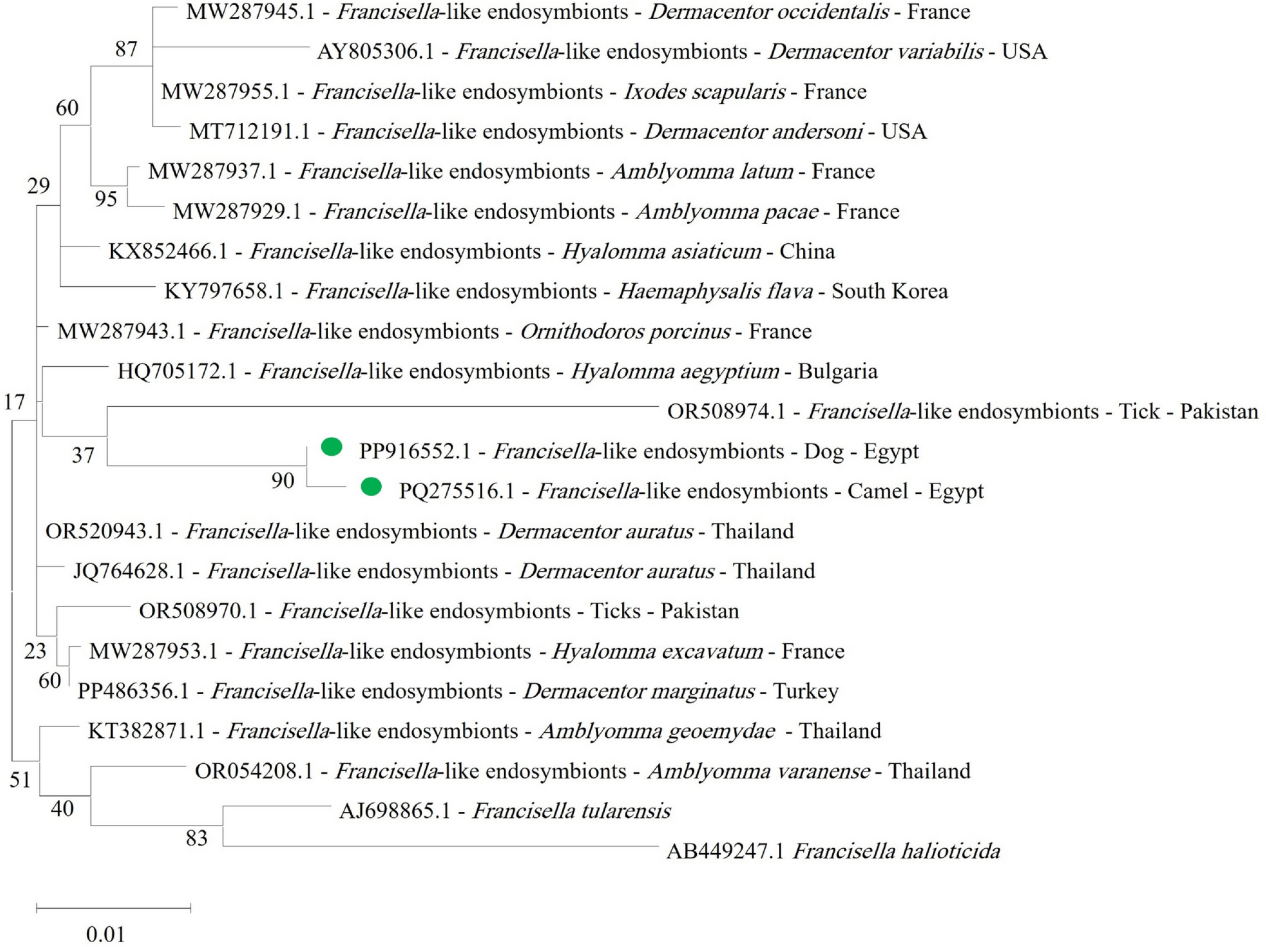
Phylogenetic relations of *Francisella-*like endosymbionts in camel and dog blood, obtained via the maximum-likelihood and the Kimura three-parameter model based on *16S ribosomal RNA* gene sequences. The percentage of trees on which the related taxa are clustered together is displayed next to the branches. Branch lengths are expressed in terms of the number of substitutions per site, and the tree is drawn to scale. The green shaded circle represents *Francisella*-like endosymbionts obtained in the present study.

**Fig. 4:**
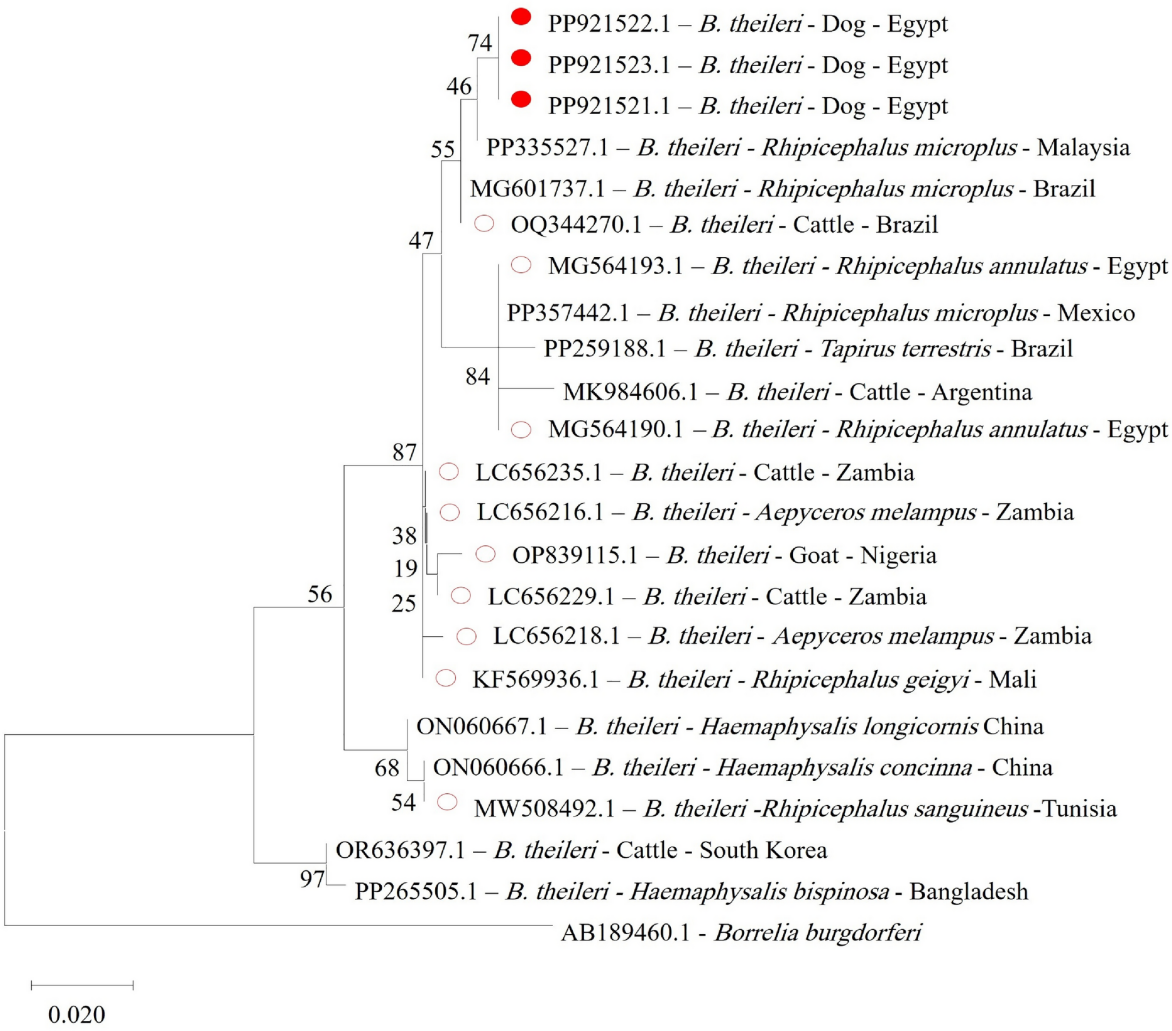
Phylogenetic relations of *Borrelia theileri* in dogs, obtained via the maximum-likelihood method and the Kimura three-parameter model based on *Flagellin B* (*flaB*) gene sequences. The percentage of trees on which the related taxa are clustered together is displayed next to the branches. Branch lengths are expressed in terms of the number of substitutions per site, and the tree is drawn to scale. The red unshaded circle related to *Borrelia theileri* isolated from different host in African country, and shaded red circles represent *Borrelia theileri* in dogs in the present study.

Sequence analysis for *Francisella*-like endosymbionts was successfully performed on two positive samples out of four. The resulting sequences were deposited in the GenBank database under accession numbers PP916552.1 for dogs and PQ275516.1 for camels. A phylogenetic tree was constructed to further define the evolutionary relationships among Francisella species (Fig. 3). For the *Borrelia theileri flaB* gene, three positive samples were sequenced out of the five detected. The resulting sequences were submitted to the GenBank database under accession numbers PP921521.1, PP921522.1, and PP921523.1, sequentially (Fig. 4). Neither Coxiella nor Bartonella species were detected in blood samples from camels or dogs in this study*.*

## Discussion

*Rickettsia aeschlimannii* has been reported to infect humans in Tunisia, Morocco, Algeria, and Greece^48–51^. *Rickettsia aeschlimannii* has been detected in ticks from Zimbabwe, Niger, and Mali in Africa^52^, as well as in Pakistan in ticks collected from camels^53^. In Nigeria, *Rickettsia aeschlimannii* was detected in 8.6% of ticks collected from camels^53,54^. In southern Egypt, *Rickettsia aeschlimannii* was detected in ticks from camels at an infection rate of 6.06%^55^. In this study, 5% of camel blood and 1% of dog blood samples were found to be positive with *Rickettsia aeschlimannii*. Several studies in Egypt have found various Rickettsia species in ticks collected from dogs, including *Rickettsia conorii* (at a positivity rate of 16.2%) and other Rickettsia species (18%)^56^, both of which are higher than the rates reported this study. Previous studies suggested that ticks can transmit *Rickettsia aeschlimannii* to camels^57^, which is consistent with the results in this study, and indicates that both camels and dogs may act as reservoirs for *Rickettsia aeschlimannii*.

Phylogenetic analysis depends targeting the *gltA* gene for five *Rickettsia aeschlimannii*-positive blood samples from camels and the single positive sample from a dog (accession number PQ306608.1) showed 100% identity with deposited sequences in GenBank for ticks from Nigeria (MN601328.1), France (OR288101.1 and PP379722.1) and Egypt (LC785431, LC785432, LC785433), and they were found in the same clusters (Fig. 2).

This study is the first report of *Rickettsia aeschlimannii* in camel and dog blood in southern Egypt, highlighting the need for further research to determine the prevalence of this pathogen in various domestic animals. Camels are well-suited to hot and arid environments, making them valuable for pastoral households engaged in milk or meat production. Camels may also be used as working animals for the drawing water, plowing, and transportation^58^. The dog is an important companion animal usually present in the owner’s household environment in a modern and urbanized society. However, they may act as reservoirs of zoonotic agents, especially after being fed on by blood-sucking, which increases the risk of zoonotic infection, either through direct contact, or through insects passing infected blood between hosts when feeding^59^. Camels and dogs may thus pose risks an infection risk to humans, for disease-causing pathogens such as *Rickettsia aeschlimannii*. Monitoring prevalence rates for this in animal populations may thus provide sentinel data which will aid the prevention of human infection.

*Francisella-*like endosymbionts reportedly has prevalences of 100% in *Haemaphysalis doenitzi* ticks in China, 32% in *Dermacentor reticulatus* ticks in Sardinia Island, and 22.5% in *Hyalomma* ticks in Anatolia^60–62^. Francisella species have been detected in Egypt in *Hyalomma dromedarii* at a rate of 6%^63^. The infection rate of *Francisella-*like endosymbionts in this study was 2% for both animal species evaluated (camels and dogs).

Based on phylogenetic analysis of *Francisella*-like endosymbionts using the *16S ribosomal RNA* gene, two positive samples (one from a camel and one from a dog) were submitted for sequencing, and the results were deposited in GenBank with accession numbers PQ275516.1 and PP916552.1, respectively. The results for the these camel and dog samples showed similarity rate of 99.96% between each other, and a maximum identity rate of 99.11% when compared with other isolates in GenBank (OR520943.1 *Dermacentor auratus* from Thailand, OR508974.1 tick from Pakistan, JQ764629.1) (Fig. 3).

The identification of *Francisella*-like endosymbionts in camel and dog blood samples underscores the importance of complete vector control strategies and regular vector-borne pathogen screening for these species. This result considered the first report on discovering and characterizing *Francisella*-like endosymbionts in the camelid and canine blood samples from southern Egypt. The pathogenicity of *Francisella*-like endosymbionts is not fully understood; however, data on their detection in this region is important for assessing potential risks to human health and implementing appropriate measures.

*Borrelia theileri* has been detected in northern Egypt, with some host species-dependent variation in prevalence. In dogs, its infection rate was found to be 0.5%^64^, while the corresponding figure in cattle was 0.43%, and that in sheep was 3.4%^65^. In this study, the infection rate of *Borrelia theileri* in camels was 0%, but that in dogs was 5%.

Phylogenetic analysis results for the *flaB* gene for dog blood samples in this study was compared to other reported isolates in GenBank. No 100% similarity could be found for any previously registered isolate. The highest similarity was 99.27%, with *Borrelia theileri* recorded in *Rhipicephalus microplus* (PP335527.1 and MG601737.1) from Malaysia and Brazil, cattle from Zambia (LC656235.1), and cattle from Brazil (OQ344270.1). Additionally, there was a 98.77% similarity with isolates from *Rhipicephalus annulatus* (MG5641190.1 and MG5641193.1) from Egypt (Fig. 4). The high specificity and conservation across strains of Borrelia species led us to use the *flaB* gene in molecular diagnostics to detect and identify Borrelia species^66^. Scanning dogs for *Borrelia theileri* may thus assist in identifying potential reservoirs, thereby preventing human infections and possibility of transmission to other host species.

In this study, we could not detect Coxiella species in blood samples from camels or dogs. This finding is consistent with previous studies in Algeria, Tunisia, and Palestine, which also did not detect *Coxiella burnetii* in any camel blood samples, which may well reflect a low bacterial load in camel blood^67,68^.

Similarly, Bartonella species were not detected in camel or dog blood samples in this study. This may reflect the fact that Bartonella species are often present in very low quantities in the bloodstream, making them difficult to detect. Additionally, Bartonella can cause intermittent bacteremia, meaning the bacteria are not always present in the bloodstream. The prevalence of Bartonella species can also vary by region, and it is possible that the prevalence is lower in southern Egypt^69^.

## Conclusion

This study provides the first report on the detection of *Rickettsia aeschlimannii* and *Francisella*-like endosymbionts in both camels and dogs in southern Egypt. Additionally, Borrelia species were detected in dogs, while other target pathogens (Coxiella and Bartonella species) were not detected. Considering the potential for camels and dogs to serve as reservoirs for vector-borne diseases, it is crucial to continue monitoring as a control measure against vector-borne diseases. Future research should focus on the ecological and epidemiological aspects of these diseases to create effective control and prevention strategies.

## Supplementary Information

Supplementary Information.

## Data Availability

Sequence data that support the findings of this study have been deposited in GenBank with the accession numbers: PQ212650.1, PQ212651.1, PQ212652.1, PQ212653.1, PQ212654.1, PQ306608.1, PQ275516.1, PP916552.1, PP921521.1, PP921522.1, and PP921523.1.
