# Supplementary material for: Molecular detection of Rickettsia aeschlimannii, Borrelia theileri, and Francisella-like endosymbionts in Camelus dromedarius and dogs in Luxor, Egypt
Source: Sci Rep. 2025 Apr 15;15:12872. doi: 10.1038/s41598-025-91530-x (PMC12000560; doi:10.1038/s41598-025-91530-x)
Supplement: Supplementary file 1 — Supplementary Information. [file 41598_2025_91530_MOESM1_ESM.pptx]

## Slide 1
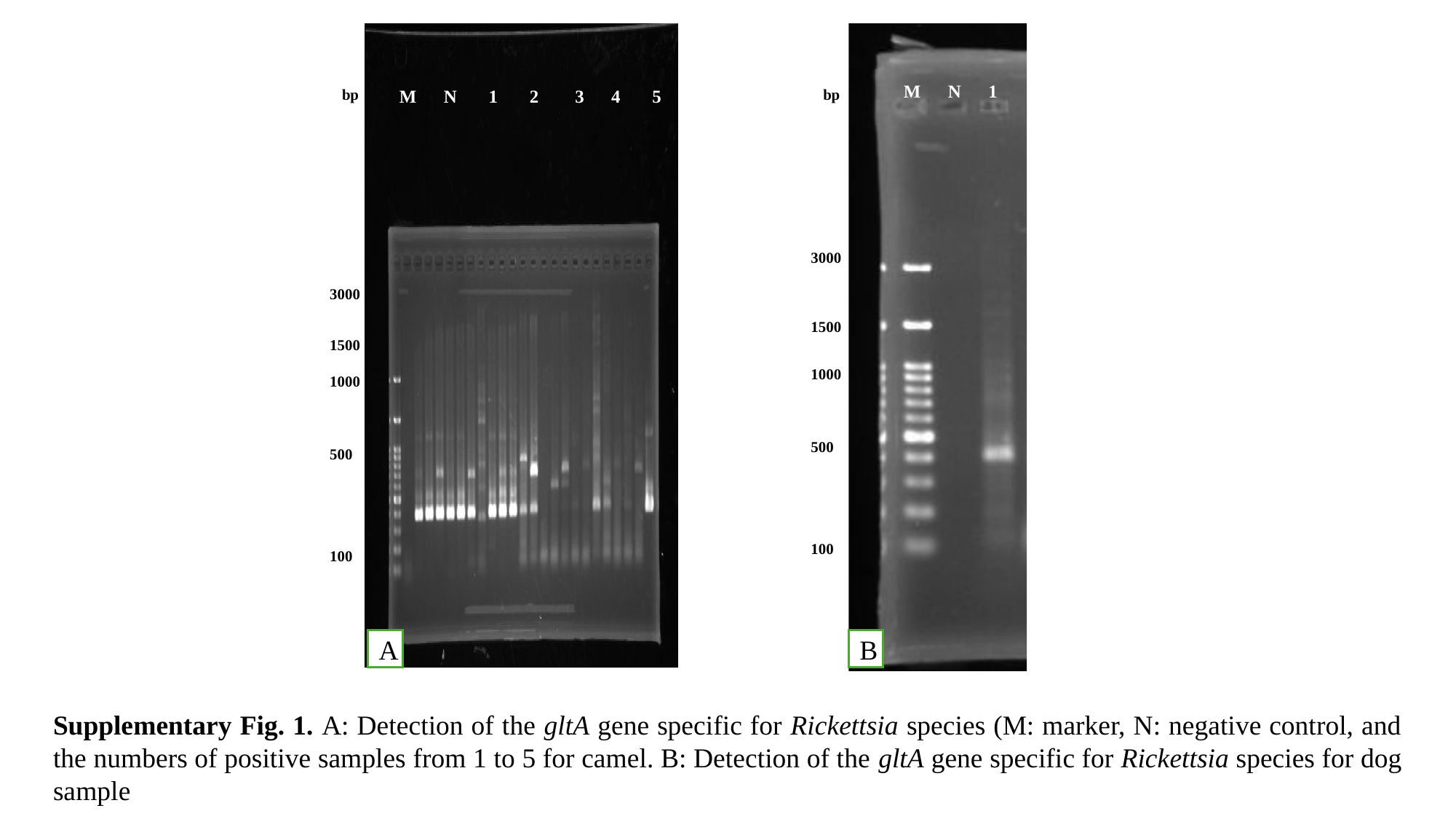

M N 1
bp
3000
1500
1000
500
100
 M N 1 2 3 4 5 6
bp
3000
1500
1000
500
100
 M N 1
A
B
Supplementary Fig. 1. A: Detection of the gltA gene specific for Rickettsia species (M: marker, N: negative control, and the numbers of positive samples from 1 to 5 for camel. B: Detection of the gltA gene specific for Rickettsia species for dog sample

## Slide 2
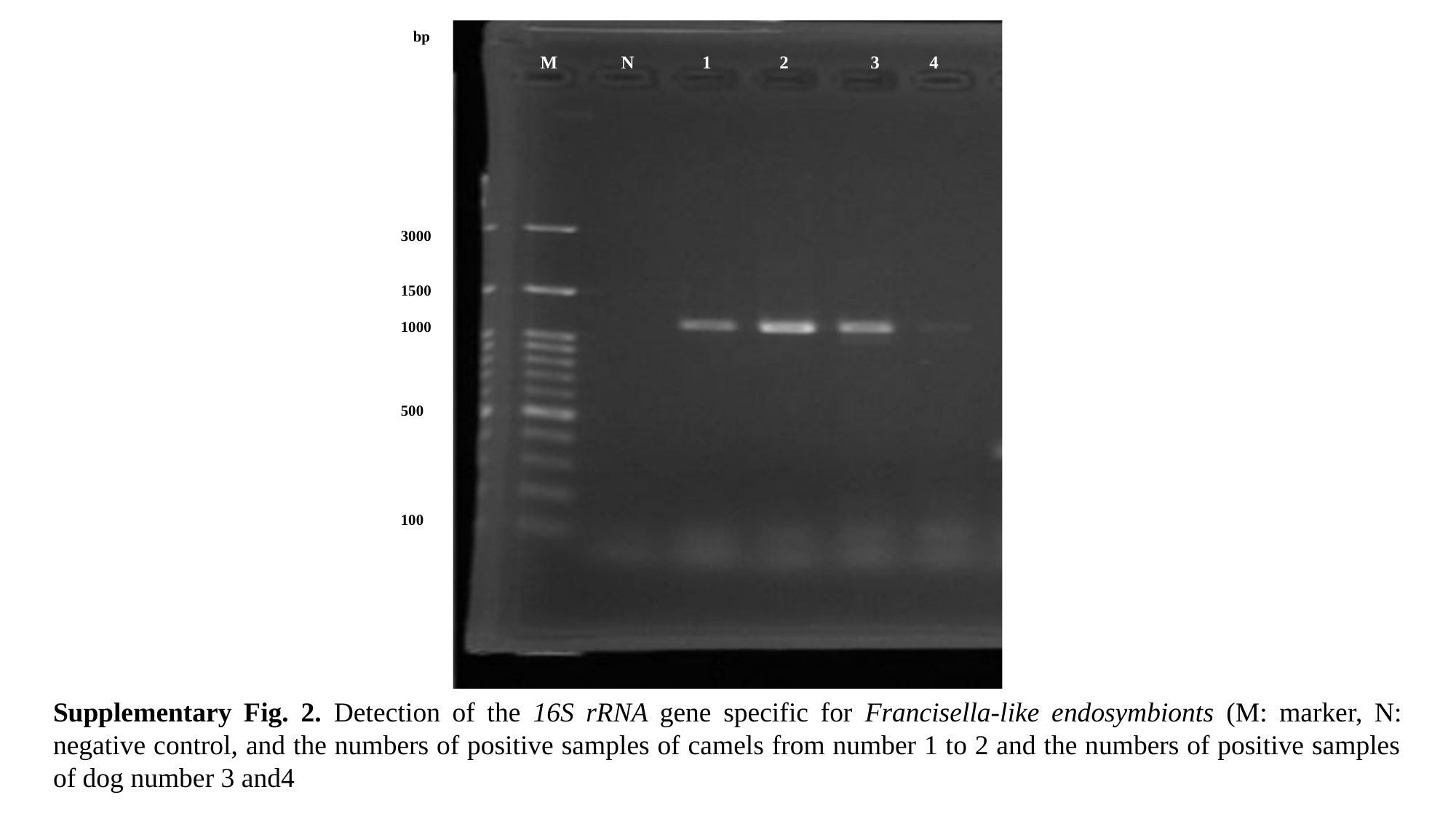

bp
3000
1500
1000
500
100
 M N 1 2 3 4
Supplementary Fig. 2. Detection of the 16S rRNA gene specific for Francisella-like endosymbionts (M: marker, N: negative control, and the numbers of positive samples of camels from number 1 to 2 and the numbers of positive samples of dog number 3 and4

## Slide 3
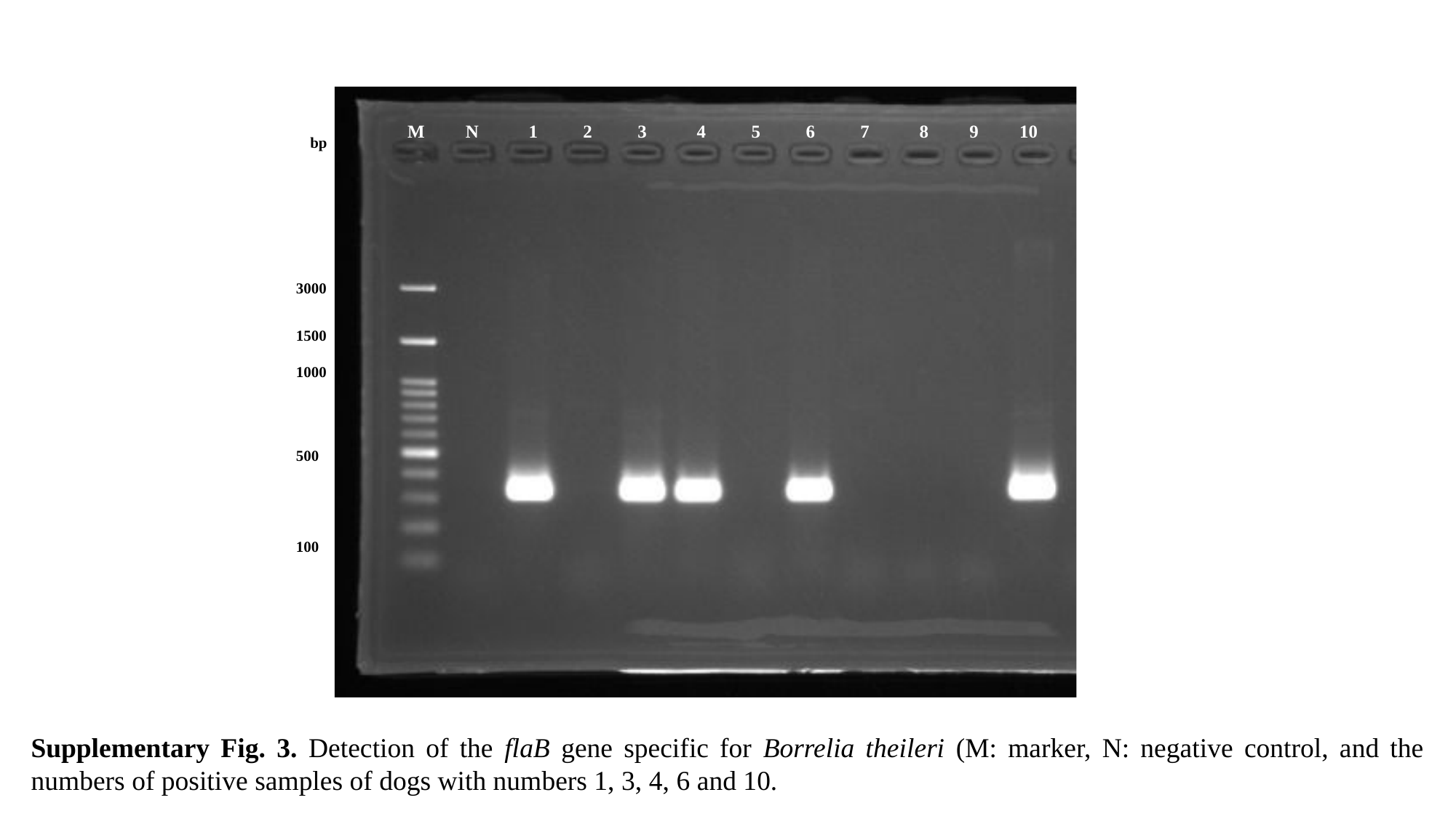

M N 1 2 3 4 5 6 7 8 9 10
bp
3000
1500
1000
500
100
Supplementary Fig. 3. Detection of the flaB gene specific for Borrelia theileri (M: marker, N: negative control, and the numbers of positive samples of dogs with numbers 1, 3, 4, 6 and 10.
